# Facilitators and barriers for implementation of a novel resuscitation quality improvement package in public referral hospitals of Nepal

**DOI:** 10.1186/s12884-023-05989-5

**Published:** 2023-09-13

**Authors:** Niina Ekström, Rejina Gurung, Urja Humagain, Omkar Basnet, Pratiksha Bhattarai, Nishant Thakur, Riju Dhakal, Ashish KC, Anna Axelin

**Affiliations:** 1 Department of Women’s and Children’s Health, Uppsala University, Uppsala, 75185 Sweden; 2 Research Division, Golden Community, Jawgal-11, Lalitpur, Nepal; 3 School of Public Health and Community Medicine, Sahlgrenska Academy, University of Gothenburg, Medicinaregatan 18 A, Gothenburg, Sweden; 4 Department of Nursing Science, University of Turku, Turku, Finland

**Keywords:** Neonatal resuscitation, Facilitation, Implementation, Plan-Do-Study-Act cycle, Nepal

## Abstract

**Background:**

Improving the healthcare providers (HCP) basic resuscitation skills can reduce intrapartum related mortality in low- and middle-income countries. However, the resuscitation intervention’s successful implementation is largely dependent on proper facilitation and context. This study aims to identify the facilitators and barriers for the implementation of a novel resuscitation package as part of the quality improvement project in Nepal.

**Methods:**

The study used a qualitative descriptive design. The study sites included four purposively chosen public hospitals in Nepal, where the resuscitation package (Helping Babies Breathe [HBB] training, resuscitation equipment and NeoBeat) had been implemented as part of the quality improvement project. Twenty members of the HCP, who were trained and exposed to the package, were selected through convenience sampling to participate in the study interviews. Data were collected through semi-structured interviews conducted via telephone and video calls. Twenty interview data were analyzed with a deductive qualitative content analysis based on the core components of the i-PARiHS framework.

**Results:**

The findings suggest that there was a move to more systematic resuscitation practices among the staff after the quality improvement project’s implementation. This positive change was supported by a neonatal heart rate monitor (NeoBeat), which guided resuscitation and made it easier. In addition, seeing the positive outcomes of successful resuscitation motivated the HCPs to keep practicing and developing their resuscitation skills. Facilitation by the project staff enabled the change. At the same time, facilitators provided extra support to maintain the equipment, which can be a challenge in terms of sustainability, after the project. Furthermore, a lack of additional resources, an unclear leadership role, and a lack of coordination between nurses and medical doctors were barriers to the implementation of the resuscitation package.

**Conclusion:**

The introduction of the resuscitation package, as well as the continuous capacity building of local multidisciplinary healthcare staff, is important to continue the accelerated efforts of improving newborn care. To secure sustainable change, facilitation during implementation should focus on exploring local resources to implement the resuscitation package sustainably.

**Trial Registration:**

Not applicable.

## Contributions to the literature

Factors such as the context, facilitation, and site of implementation influence the successful implementation of the resuscitation bundle.

The introduction of innovation for quality healthcare requires adequate facilitation and continuous capacity-building efforts.

## Background

In 2019, 2.4 million newborns died in the world within the first 28 days of life, which is in the neonatal period [1]. Low- and middle-income countries are highly represented in the number of newborn deaths, as approximately 99% of the deaths occur in these countries [2–5]. In low- and middle-income countries, intrapartum-related complications account for one-third of newborn deaths [5]. It has been estimated that 10–15% of full-term newborns need assistance to start breathing at birth, and they can require neonatal resuscitation at some level [6, 7].

The American Academy of Pediatrics developed a neonatal resuscitation program, ‘‘Helping Babies Breathe (HBB)’’ in 2010 for low and middle income settings, which was updated in 2016 [8, 9]. HBB program builds the competency of health care providers on neonatal resuscitation and improves the health facility readiness for managing high risk newborns in low resource settings [10]. HBB program aims to improve performance by early initiation of ventilation within 60 s after birth (golden minute after birth) and effective ventilation (chest rise with each ventilation). The program has been implemented in more than 80 countries and reached more than a million healthcare providers [11, 12].

A systematic review on the HBB program’s impact reported a decrease in fresh stillbirths by more than one third (34%, and neonatal mortality within the first 24 h after birth decreased by one third (30%) [13]. Systematic review(8) has shown several barriers for HBB program’s to early initiation of ventilation within first 60 s (Golden minute) i.e. health facility set up i.e. distance between the delivery bed and resuscitation table, limited time devoted to frequent training and practice, staff turnover after HBB training, and the price and maintenance of equipment [14]. This indicates successful implementation of new intervention and technology requires understanding of the context of health facility and facilitate to introduce the interventions in the facility and among health care providers.

In 2019, Nepal recorded 11,128 neonatal deaths [15]. Although the numbers have continued to decrease since 1960, Nepal still has high neonatal mortalities with a neonatal mortality rate (NMR) of 19.8 per 1,000 live births in 2019 [16]. The HBB program was implemented in a stepped-wedge cluster randomized controlled trial to evaluate a quality improvement package for neonatal resuscitation on intrapartum-related mortality in Nepal during 2017–2018 [17]. The results suggested that HBB can reduce intrapartum-related mortality and, potentially, early neonatal deaths in the Nepalese context [18]. A better understanding of the implementation process is necessary to support the implementation fidelity and sustainable implementation of the resuscitation bundle. The purpose of this study was to identify some of the facilitators and barriers for the implementation of the resuscitation bundle as a part of the Scaling Up Safer Bundle Through Quality Improvement In Nepal (SUSTAIN) project.

## Methods

### Study design

The study used a qualitative descriptive design. The study sites included four Nepalese hospitals where the SUSTAIN project had been implemented. For 2019–2021, the SUSTAIN project was conducted in eight public Nepalese hospitals [19]. Each of these hospitals have at least 3,000 deliveries a year, and the resuscitation bundle was implemented in these eight hospitals as a part of the project. The SUSTAIN project had a study period of 21 months in each hospital with implementation and intervention periods. The study interviews were conducted between October 2020 and May 2021.

### Implementation of the resuscitation bundle

To improve the quality of neonatal resuscitation, the SUSTAIN project provided each hospital with the HBB guidelines and the following new equipment: upright bag-masks, NeoBeat newborn heart rate monitors, and NeoNatalie live training manikin. In each hospital, before introduction of neonatal resuscitation package, external facilitators together with hospital team conducted context analysis of the maternity care and planned for introduction of resuscitation training, quality improvement package, equipment and continuous measurement of resuscitation. Following which training on HBB was conducted to all health care providers working in the maternity ward. To measure the performance of resuscitation, observation of the resuscitation care was done independently and recorded in progress board on a daily basis. The measure of resuscitation included ventilation within golden minute and ventilation at rate of 40 to 60 bpm. A bi-weekly quality improvement meeting using Plan-Do-Study-Act (PDSA) was established by the external facilitator and hospital leadership to review the progress in neonatal resuscitation and review the data based on progress board measure. The skill-drills in the high fidelity neonatalie were conducted on daily basis to maintain the resuscitation skills in the maternity ward. The project staff facilitated the implementation and was involved in the daily practice, data collection, and management.

SUSTAIN project adapted the HBB protocol with use of NeoBeat, where in all newborns who do not cry at birth, stimulation was to be done. The newborn was then assessed for breathing, if there was no or difficulty in breathing, secretion to be assessed. If there was any secretion, suction was to be done to remove the secretion. The newborn was further assessed for breathing, if there was no/difficulty in breathing, ventilation was to be initiated. After first ventilation, the breathing was assessed together with HR, the HR was measured using NeoBeat. Both breathing and HR was used to guide further ventilation procedure.

### Participants

Four hospitals were sampled purposively by using maximum variation sampling [20]. Convenience sampling was used to select participants for interviews. To gain enough variation, two hospitals, for which the SUSTAIN project implementation had not been very successful, and two hospitals, for which the implementation had been successful, were invited to participate. In the four selected hospitals, the inclusion criteria for eligible health care providers (nurse-midwives and nurses) working at the labor and maternity units were the following: (1) had worked at the ward during the entire SUSTAIN project; (2) worked closely with the mothers and babies; and (3) participated in the skill-drills, skill checks, and PDSA-meetings. All eligible health care providers (nurse-midwives and nurses) in the four hospitals received invitation emails to interviews. Informational redundancy within the deductive analysis framework started to appear after 12 interviews [21]. To secure data saturation two more interviews were conducted in each study hospital. In total 20 participants were included in the study. All participants were females with a mean age of 34 (range 24–52). They all had different levels of nursing or midwifery education, and their mean years of practice was 12 years (range 1–27).

### Data collection

The research team facilitated and undertook individual semi-structured interviews via telephone and video calls for data collection. During the interviews, participants were asked to describe and reflect on their experiences about the subject. Further information was elicited with follow-up questions e.g., Why did it (not) work?; Could something be done differently? The Nepalese interviewers were male and female with previous experiences with qualitative research and interviews. Some also had clinical backgrounds, for example as medical doctors, midwives, or public healthcare nurses. The interviewers and participants had previously worked together in the SUSTAIN project. A semi-structured interview guide was developed to inquire about participants’ perceptions regarding potential changes in resuscitation practices and to recognize the facilitators and barriers for implementing the HBB and the new equipment (Table 1). The i-PARiHS framework (integrated Promoting Action on Research Implementation in Health Services) was used as a theoretical framework to interpret implementation outcomes retrospectively. The research team developed the original interview guide in English and then translated it to Nepalese. The translation’s congruence was secured by the discussion within the research team, and the interview guide’s functionality was tested with two pilot interviews. Small modifications were done to the interview guide, so that the questions were more open to elicit participants’ perceptions. The pilot interviews were conducted in hospital which was not part of this study, and the data of the pilot interviews was not included in the analysis.

**Table 1:** Interview Guide for Data Collection

Gender and profession of the interviewer:
Introduction questions: – Gender: – Age: – Profession and education: – Years of practice: – Hospital and ward:
1. What do you know about the SUSTAIN project? Can you briefly explain it?
2. Describe your current practices in neonatal resuscitation.
3. What new has the SUSTAIN bundle brought to your practice?
4. What have been your experiences on (1) NeoNatalie live training and (2) using the upright newborn bag-mask for neonatal resuscitation? (Innovation) – Follow-up questions: Why it worked? Why it did not work? Your own thoughts on the importance of training and using the bag-masks? (recipients, motivation, goals, skills, etc.)
5. What have been your experiences using NeoBeat Newborn Heart Rate meter? (Innovation) – Follow-up questions: How have you used it? Why it worked? Why it did not work? Your own thoughts on the importance of using NeoBeat? (recipients, motivation, goals, skills, etc.)
6. How did you experience the SUSTAIN project’s two-month training period? (1) Facilitators from the research staff and your own hospital, (2) PDSA-meetings and (3) skill drills? (Facilitation) – Follow-up questions: What was helpful? What was not helpful? What could have been done differently to facilitate trainings and, thus, the implementation?
7. How did you experience the leadership? Have the leaders visited the unit/ward? Have you discussed SUSTAIN with the leaders? and How did you experience other resources during the SUSTAIN project? Have you received extra resources from the SUSTAIN project for your work? (context) – Follow-up questions: Have you received any feedback from the leadership during the process? How was the teamwork? Has the COVID-19 pandemic impacted the implementation?

### Data analysis

The data were analyzed simultaneously with the data collection. The interviews were recorded, transcribed in Nepalese, and translated to English. After completion of two interviews, the English translations were immediately coded to check data saturation. The data collection continued until the information redundancy was reached [22]. The analyzing process followed qualitative content analysis with the deductive approach described by Elo and Kyngäs [20]. The interview transcripts were read thoroughly, and one researcher (NE) coded the data. The text was coded under five deductive categories from the semi-structured interview guide: ‘‘practice change’’ and the four core constructs of i-PARiHS framework (i.e., innovation, recipients, facilitation, and context) [21]. After the codes were grouped under five categories of the deductive framework, sub-categories were developed following the principles of inductive content analysis to create the findings under each five categories. AA closely supervised NE throughout the analysis. The supervisor also familiarized herself with the interview transcripts. All disagreements in the analysis were solved with discussions.

### Ethics

The SUSTAIN project received an ethical approval from the Ethical Review Board of Nepal Health Research Council (number-110/2019), and the participating hospitals approved the study. All participants gave a written informed consent via email, and verbal consent was recorded before the interview. Interview data were anonymized, stored, and handled confidentially.

### Findings

The findings suggest that there were changes in the old resuscitation practice among the staff, as well as the introduction of more systematic and effective practice after the SUSTAIN project’s implementation. Different factors assisted the positive changes, and there were barriers to the implementation.

### Practice change – move to a more systematic resuscitation

According to the health care providers, the HBB implementation clearly changed the resuscitation practices in each of the four participating hospitals. The health care providers described that, before the SUSTAIN project, there was no regular training on neonatal resuscitation. Tube suctioning was done to all newborns, and when necessary, ventilation was provided through a horizontal bag-mask with an oxygen fitting system. The initiation of resuscitation was often delayed due to the lack of a clear resuscitation protocol and a determination of the newborn’s heart rate. To determine whether a newborn had a pulse, the health care providers used manual palpation and a stethoscope. These procedures required at least two staff members, and they interrupted ventilation as one health care provider described: *“I mean, in the past, when we did bag-and-mask we had to put the stethoscope in the ear and listen to the heart rate … we had to look at the time … we had to stop working for a while and listen to the heart rate’’* (ID06).

After the SUSTAIN project, all four sites performed neonatal resuscitation mainly in line with the new protocol. The nurses described that resuscitation had become more systematic and that it was initiated earlier and performed longer. NeoBeat helped the nurses to identify the newborns requiring resuscitation and distinguished them from fresh stillbirths. Among the non-breathing newborns after stimulation, health care providers often successfully initiated ventilation within 60 seconds of birth (golden minute). The NeoBeat displayed the heart rate during ventilation and help guide ventilation, while previously after the first ventilation, health care provider had stopped the ventilation procedure to check for heart rate either by cord pulsation or stethoscope. NeoBeat enabled continuous heart rate monitoring and continue ventilation until the newborn was stabilized: “*Now when we resuscitate, we don’t even have to listen with the stethoscope … once we put it on (NeoBeat), it instantly shows the heart rate … we can also determine if effective ventilation has been done or not and how long shall we continue’’* (ID10). The health care providers performed ventilation by using the upright bag-mask, although some of the hospitals still used the old horizontal bag.

### Innovation – neonatal heart rate monitoring driving the change

There was a high demand for the new equipment. According to the health care providers, all new devices were in continuous use. The provided equipment made work easier, which encouraged the nurses to use it: “*The equipment provided by SUSTAIN has made it very comfortable and easier to work’’* (ID08). Theory and knowledge behind each equipment was also seen as a motivating factor to use them. The health care providers considered penguin suction to be good, helpful, and less risky than the old suctioning device. The upright bag-mask was easier to use and more ergonomic than the old horizontal bag-mask. Improved ventilation was provided with the new bag-mask, and the health care providers experienced it was safer for the newborns because there was not much leakage.

The health care providers explained that NeoBeat had contributed to the biggest change in their practice, as it guided resuscitation by instructing whether ventilation should be initiated, how long it should be provided, and if a doctor should be called. NeoBeat saved time, required fewer staff resources, and therefore, helped the nurses to reach the golden minute. The health care providers considered the NeoBeat reliable and effective. They also explained that the doctors trusted NeoBeat, which led to better communication between the professions: “*Using NeoBeat has made resuscitation much easier, it has made heart rate monitoring very convenient, and the reading provided by it is better than that obtained from listening to the stethoscope*” (ID14).

The health care providers indicated that one of the biggest barriers for the effective implementation of HBB was the lack of equipment, which led to iniquity in patient treatment. Some of the equipment was damaged during sterilization, and the amount of provided equipment was limited. In some hospitals, an old oxygen system has hindered practice changes in ventilation. The old horizontal bag-masks were preferred because they had an oxygen fitting system, and the old resuscitation routine included the use of oxygen: “*We have another horizontal bag-and-mask with oxygen fitting system and prefer to use it more frequently’’* (ID03). Technical difficulties with NeoBeat also caused a barrier for its use. For example, it caused confusion by showing a heartbeat on a newborn with no breathing and heartbeat confirmed by stethoscope after prolonged ventilation and was declared deceased. In addition, not all nurses were trained to maintain the NeoBeat. The lack of systematically implemented maintenance led to situations in which the health care providers did not remember to charge the device.

Recipients – *Feedback supporting the change*.

The nurses explained several motivating factors, such as reaching the set goals and seeing the positive outcomes that supported them, to continue practicing and developing resuscitation practices. The nurses succeeded in resuscitating the newborn and reaching the golden minute. The health care providers reported to have gained confidence in the training with NeoNatalie and saw improvement in their own skills. This motivated the health care providers to continue perfecting the skills through training. The health care providers learned that teamwork had been good during the project: *“We perform all these activities in team including project staff that we have been provided*’’ (ID06). Trust was established between the hospital and project staff, and everyone’s input affected the outcome as the context analysis of the maternity care in the hospital was done together as team during the introduction of the intervention package. The health care providers attitudes and trust toward the intervention package gradually improved, which led to better performance visible in the progress board during training and daily work.

At the same time, some of the health care providers explained that not all staffs were motivated to train resuscitation. Some of the health care providers felt that their colleagues were negligent toward the new routines and equipment, which they thought was irresponsible: “*The main barrier is negligence from hospital staffs because although the delivery decreased by half but still, they are not performing skill drill”* (ID01). In addition, the health care providers brought up the lack of staff multiple times, and they explained that teamwork was harder when there were not enough nurses on duty. They did not have time to do all their normal tasks, and the project increased workload because the new routines included more steps, skill drills, trainings, and meetings that required time. In addition, there was some confusion between the professions because only the health care providers took part in the SUSTAIN trainings, and the doctors did not share the same information.

Facilitation – *An enabler for change and a barrier for sustainable change*.

The health care providers described that the facilitators and SUSTAIN project staff provided by the project were effective and that the trainings were informative. The health care providers positively experienced putting theory into practice. New things were learned, and updated information was provided to refresh knowledge. The health care providers explained that regular training had led to new routines, which made daily practice easier, and continuous practice helped retain skills. Successful trainings motivated the health care providers to practice more. However, the health care providers indicated that the skill drills were done outside the working hours, which meant that they did not always have the time to practice and were exhausted: *“Due to the lack of staff we had to work on night duty and attend the training in the morning hour, so the schedule was so packed that we were exhausted’’* (ID06).

The health care providers also perceived the facilitators as nice, talented, and positive. They explained that the project staff had provided human resources and equipment. The project staff worked at the wards and was involved in the daily practice, making the health care providers’ work easier. The health care providers also noted that the project staff took care of the equipment by cleaning and charging the units. In addition, they had regular follow-ups with the nurses and encouraged them to change the practice: “*Yes, we are getting more support than before on our work in aspects such as human resources, equipment, management, PDSA meetings, feedback and so on*” (ID05).

The health care providers explained that PDSA meetings were held biweekly. The data showed at the meeting illustrated how many times the equipment was used, how many times the golden minute was achieved, and how well the trainings went. The health care providers discussed the problems, and new solutions were introduced. The PDSA meetings improved daily practice, led to skill improvement, motivated the health care providers, and provided transparency to the implementation process: “*PDSA has helped to solve common gaps and problems, change our behavior and improve our clinical practice*” (ID03).

Context – *The unclear role of leadership and medical doctors*.

The health care providers explained that most of the feedback supporting practice change was received in the PDSA meetings. This constant feedback for the health care providers led to improvement in daily practice. The health care providers also received feedback from a tablet that recorded their performance in the trainings: “*Intrapartum care has improved through constant feedback and support from mentors, their facilitation and mostly because of SUSTAIN program itself*” (ID06). The leadership and management for the change was perceived good. The health care providers indicated that coordination and delegation from the leaders had worked well. They explained that the department head and chief of nursing visited the ward every day to observe the practice. However, some of the health care provider seemed to be a bit reserved when answering questions about the leaders and management. One health care provider also noted that the hospital management was poor and that the leaders could have played more active roles in supporting the project. The health care providers said that there had been dysfunctional interprofessional communication. At some hospitals, only the chief of nursing discussed the protocol with the ward staff. The project staff was available for questions, but due to the busy schedules, the support was sometimes inhibited. The health care providers found that they were trusted, although they also indicated that sometimes teamwork was ineffective: “*We have to follow the doctors rather than our practical knowledge, that’s a problem we are facing*” (ID09).

## Discussion

The study recognized the major facilitators and barriers of implementing the resuscitation bundle and assisting technology as a part of the SUSTAIN project through the i-PARiHS framework. The main facilitators were the provided equipment (i.e., heart rate monitor, upright bag-mask, and live training manikin), increased confidence, the positive results of the implementation, facilitation by the project team, and the feedback and discussions received during the PDSA meetings. Whereas the project team’s strong involvement in the daily practice, lack of resources, negative attitudes toward resuscitation training, unclear role of leaders, old routines, and lack of multidisciplinary commitment were seen as the main barriers.

Previous studies have shown that the HBB program’s implementation has improved the quality of neonatal resuscitation and decreased the number of neonatal mortalities and fresh stillbirths [23–25]. Health care providers in our study also described how their resuscitation practices were more systematic after the implementation the SUSTAIN project. Specifically, the continuous electronic heart rate monitor was mentioned as a driver for improved practice, as easy heart rate monitoring supported faster initiation of ventilation. Heart rate is a sensitive indicator of the newborn’s condition, and continuous heart rate monitoring during neonatal resuscitation is more accurate than using a stethoscope or manual pulse palpation [26, 27]. Immediate heart rate monitoring is extremely important as it guided ventilation and reduced the time to manually measure the heart rate using cord pulse and stethoscope, which helps for continuous ventilation [28]. Our findings strongly support the implementation of continuous electric heart rate monitoring as part of the HBB program. However, the accurate use and maintenance of the equipment are essential elements of successful implementation [14, 29].

A previous study recognized hands-on practice and increased staff confidence as facilitators [23]. The nurses in this study also gained confidence in their daily practice after trainings and skill drills. However, leadership should include training as part of the nurses’ daily work. This training would improve their motivation and signal that high quality newborn resuscitation is a priority of the hospital [29]. The other important responsibility for leadership is to provide feedback about the implementation efforts to the staff [30]. In the SUSTAIN project, the project staff provided feedback during PDSA meetings. In open discussions with nurses, the staff developed solutions on how to improve daily practice. This secured the HBB adaptation in the local context by making practice changes manageable and reasonable [31]. For sustainable practice change, leadership should also adopt this activity as part of their management strategy [32].

Facilitation by the project staff was key to improved resuscitation practice in our study. Project staff was involved in the daily practices, trainings, PDSA meetings, and equipment maintenance. The sites relied heavily on facilitation, especially through human resources. Facilitation was a proven and effective implementation strategy because it makes complex changes tangible and shares the responsibility and decision-making about implementation with the staff [33, 34]. However, in our study, it seemed that facilitators supported the staff for implementation of the resuscitation bundle and motivated them to initiate the new routines. Strong facilitator responsibilities regarding implementation efforts can challenge sustainable change.

Maintaining skills, especially the ones that are not used frequently, such as bag-and-mask ventilation, after the implementation is crucial for sustainability and for reaching the sustainable developmental goals by 2030, and refresher trainings are needed to achieve this [36]. As seen in previous studies, knowledge and skills decrease with time unless frequent training and refresher training is held [18, 19]. Therefore, frequent trainings and retesting of skills should be implemented as continuous education for all health care providers and in the orientation of new health care providers.

### Strengths and limitations

The credibility of the findings is supported by data saturation as it made it possible to provide credible answers to the research questions [35]. The study population included participants from successful and unsuccessful sites to capture variation in implementation success. However, selection bias might have occurred, as health care providers with positive views are more likely to participate. In addition, the semi-structured interview guide and sometimes leading interview technique have limited the participants to express their experiences. Our findings highlighted the positive aspects of the SUSTAIN project. However, we found some important barriers for HBB implementation. During the analysis process, some nuances of the interview data might have been lost in the translation from Nepalese to English. The authenticity and truthful interpretation of data was maintained with continuous reflective discussion between researchers in Nepal and Sweden. The facilitators and barriers that were found in this study are site-specific, and their transferability to other contexts should be applied critically.

## Conclusions

This study has identified some key facilitators and barriers for the SUSTAIN project’s implementation. The sites where the SUSTAIN project was implemented have mostly benefited from the project and a practice change, and positive effects have been seen in the study hospitals.

The Sustainable Development Goal 3.2 aims to improve the survival of newborns for which improving the care and services of high-risk newborns will be critical and will require innovations in technology and quality improvement approaches. This study provides evidence that introduction of innovations to improve resuscitation care for high-risk newborn requires facilitation using Plan-Do-Study-Act approach, understanding of the context and, and transferring the skills to healthcare providers to a sustainable care.

## Data Availability

The datasets generated and/or analyzed during the current study are not publicly available due to the involvement of information of individuals disclosing identifiers, but are available from the corresponding author on reasonable request.

## Abbreviations

SUSTAIN: Scaling Up Safer Birth Bundle Through Quality Improvement in Nepal
HBB: Helping Babies Breathe
SDG: Sustainable Development Goal
NMR: Neonatal mortality rate
PDSA: Plan-To-Do-Study-Act
i-PARiHS: Integrated Promoting Action on Research Implementation in Health Services
